# A Perioral Soft Tissue evaluation after Orthognathic Surgery Using Three-Dimensional Computed Tomography Scan

**DOI:** 10.2174/1874210601812010366

**Published:** 2018-04-30

**Authors:** Rahul Tiwari, P. Srinivas Chakravarthi, Vivekanand S. Kattimani, Krishna Prasad Lingamaneni

**Affiliations:** Department of Oral and Maxillofacial Surgery, Sibar Institute of Dental Sciences, Guntur, Andhra Pradesh, India.

**Keywords:** Assessment, Esthetic surgery, Perioral tissues, Prediction, 3D Analysis, Three Dimensional Computed Tomography (3DCT)

## Abstract

**Background:**

Facial appearance is an important factor, affects social and psychological well-being. The ideal positioning of jaws and soft tissues is crucial during orthognathic surgery for a better outcome, but the response of facial soft tissues does not always reflect the exact movements of the underlying jaws in 1:1 ratio. So, soft tissue changes following orthognathic surgery require utmost attention during surgical correction to make successful treatment.

**Aims and Objectives:**

Evaluation of perioral soft tissue changes after orthognathic surgical procedures. The objectives of the study were to assess and compare pre and post-operative perioral soft tissue changes of lip width, nasolabial and mentolabial angle using Three Dimensional Computed Tomography scan (3DCT).

**Patient and Methods:**

The study involved ten patients for evaluation requiring orthognathic surgical procedures (maxillary or mandibular anteroposterior excess or deficiency, transverse deformities, vertical maxillary excess and facial asymmetry) presented to the department of oral and maxillofacial surgery during 2014-2016. Pre and post-operative 3DCT scan were taken after 12 months using iCT 256 slice whole body CT scanner and evaluated for changes using Dicom PMS D view.

**Results:**

Significant changes were observed in nasolabial angle after maxillary advancement (1.81°) and maxillary setback procedure (2.73°). The mentolabial angle was significantly increased with mandibular setback procedures (3.27°). Mandibular advancement procedures showed both increase (3.6°) and decrease (7.6°) in mentolabial angle.

**Conclusion:**

3DCT showed a significant difference in perioral soft tissue changes in nasolabial and mentolabial angle but no significant change was observed in lip width. 3DCT is a reliable tool for 3D assessment. The conventional thought of changes in Nasolabial angle after surgery is changing due to the underlying factors which should be considered for prediction.

## 1 INTRODUCTION

Orthognathic surgery is the hallmark procedure for the correction of jaw function and esthetics of the face. The response of soft tissues always does not reflect the exact movements of the underlying jaws in 1:1 ratio [1]. Several soft tissue changes will occur following skeletal repositioning of the face which requires attention. The need of each patient will be fulfilled through some form of corrective surgery (single or bi-maxillary surgeries associated with augmentation, reduction and soft tissue surgery like rhinoplasty). The goal of orthognathic surgery is to achieve balanced occlusion and good facial aesthetics [1]. Facial esthetics has become a very important objective of Orthognathic surgery. It is of utmost importance to properly analyze and correctly diagnose the case for best treatment planning to achieve a better prognosis. As we perform the movements of jaws during surgery in 3 dimensional then why to perform the pre-operative work in 2 dimensions. In this context, 3-D imaging is the best way available for treatment planning which gives accurate measurements in anteroposterior, superoinferior and mesiodistal planes. Prediction tracing, mock surgery and post-operative analysis performed in 3 Dimensional software give us a better understanding of the case as it gives the whole positive replica of the jaws. The bony movements performed during these various orthognathic procedures by maxillofacial surgeons contemplate in the facial soft tissue [2]. Analyzing the hard and soft tissues of the face in three dimensions is needed to achieve good post-operative results [3, 4]. Two-dimensional analysis by radiographs and cephalometry have its own limitations for 3D assessment. When the two-dimensional evaluation is performed, it gives the data in only 2 axis but the 3-dimensional study of anything provides us the data in all the 3 planes. Available published literature regarding 3-dimensional analysis and post-operative changes after Orthognathic surgery is available only from the west and very few from Asian countries and same data is scarce in India too [5]. So, this study was planned to assess perioral soft tissue changes after orthognathic surgery using 3DCT scan.

### 1.1 Aim

The aim of the study was to evaluate and compare changes in lip width, nasolabial and mentolabial angle after the orthognathic surgical procedure using Three-Dimensional Computed Tomography scan (3DCT).

### 1.2 Patients and Methods

The patients visited for assessment of sleep apnea study with 3DCT volumetric airway assessment requiring orthognathic surgical procedures were randomly involved for assessment of perioral soft tissue changes. Total of 10 (4 males and 6 females) patients with age range of 18 to 26 years willing to participate and consent for use of their data for assessment in the study protocol have been enrolled in the department of oral and maxillofacial surgery during the year 2014-2016. Patients involved in the study are allotted with a lottery method because of the duration of course of study and longer duration of follow up which has restricted us to involve more number of sample size. Even though the sample size is small, the study is giving relevant and important information regarding three-dimensional changes in the series of 10 patients. The study protocol was approved by the Institutional ethics committee on 18/12/2014 (Reg. No. D148502044). Patient and relatives had been explained about the surgical procedure involved with the post-operative protocol. The informed written consent was obtained. The previous results and prediction were based on 2D but in the recent past 3D assessment has started. In this context, the dynamic tissue movement is relevant as shown in the Video no. 1

### 1.3 Inclusion and Exclusion Criteria for Patient Selection

The patients reported with facial asymmetry, maxillary and mandibular prognathism and retrognathism were included in the study and systemically compromised, drug or alcohol abuse, psychologically ill, current or past radiotherapy and patients who are not willing to enroll in the study for proper follow up were excluded.

## 2 METHODOLOGY

### 2.1 Assessment Procedure: Points to be Measured

#### 2.1.1 Nasolabial Angle

**Fig. (1):**
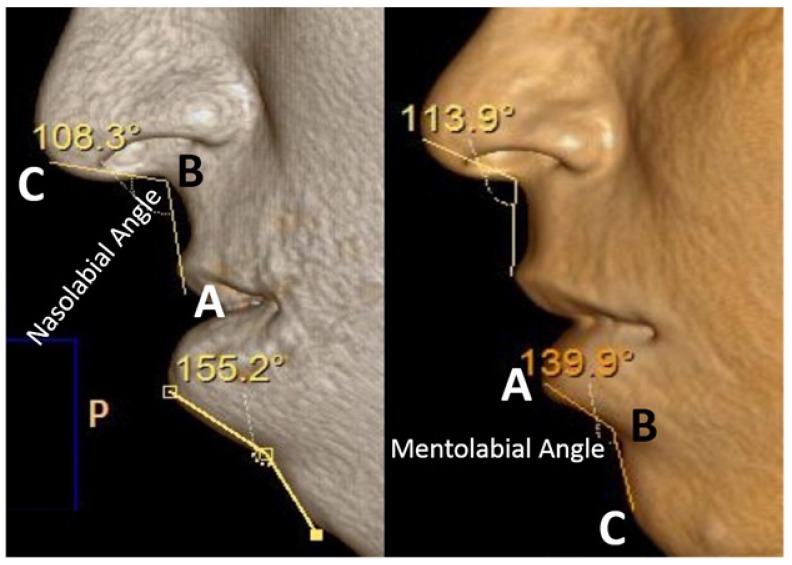
Pre and post-operative close up profile 3DCT picture showing assessment of nasolabial and mentolabial angle.

It is the angle constructed between Columella lobular junction (Cl), Subnasale (Sn), and Upper Lip (UL) [6] (Fig. **1A**).

- UL- Upper Lip- It is the most anterior point of the vermillion border of Cupid’s bow of upper lip [7].
- Sn- Subnasale- It is the point at which columella meets with an upper lip in sagittal plane [7]
- Cl- Columella lobular junction- the junction between UL and Sn [7].

#### 2.1.2 Mentolabial Angle

Angle constructed among Lower Lip (LL), Soft Tissue B Point (B), and Soft Tissue Pogonion (Pog) [6] (Fig. **1B**).

##### 2.1.2.1 LL- Lower Lip

It is the most prominent point of the vermillion border of Cupid’s bow of lower lip [7].

##### 2.1.2.2 Pog- Soft Tissue Pogonion

 It is the most prominent point of the chin [7].

##### 2.1.2.3 B - Soft Tissue B Point

 It is the most concave point of the curve between LL and Pog [7].

### 2.2 Lip Width

It is the distance between Cheilion of the Right Side (Rt.Ch) and the Cheilion of the Left Side (Lt.Ch) [6] Fig. (**2**).

#### 2.2.1 Rt.Ch- Right Chelion

It is the most lateral extent of the outline of lip on the right side [7].

#### 2.2.2 Lt.Ch- Left Chelion

It is the most lateral extent of the outline of the lip on left side [7].

**Fig. (2):**
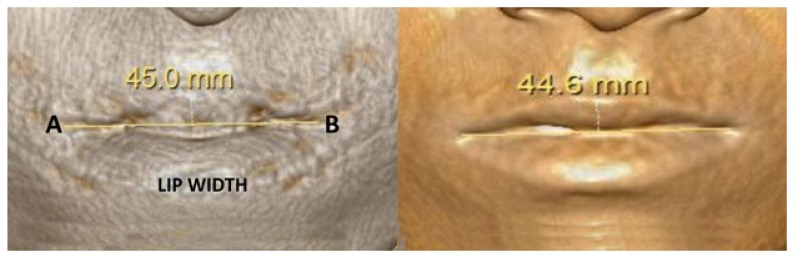
Pre and post-operative close up frontal 3DCT picture showing assessment of lip width.

**Fig. (3):**
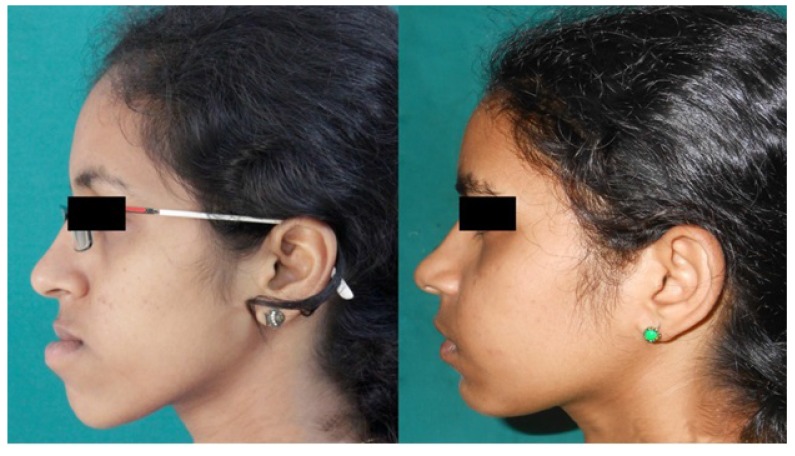
Pre and post-operative profile view of the patient – right side.

**Fig. (4):**
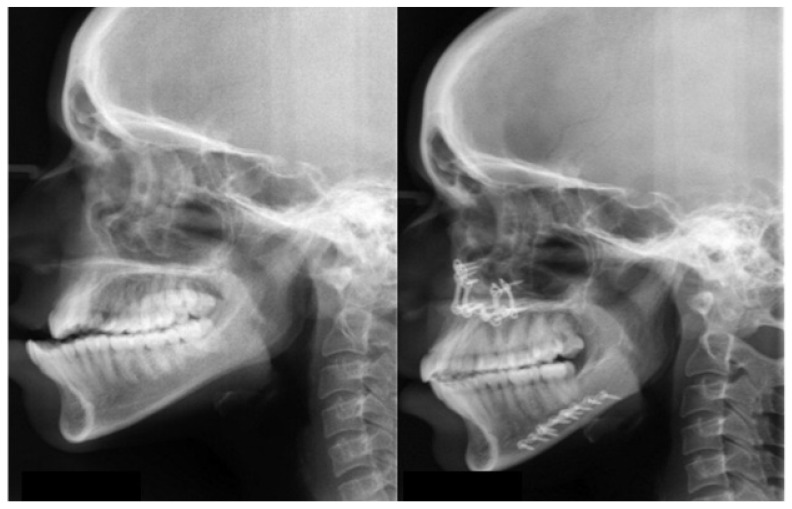
Pre and post-operative lateral cephalogram of the patient.

**Fig. (5):**
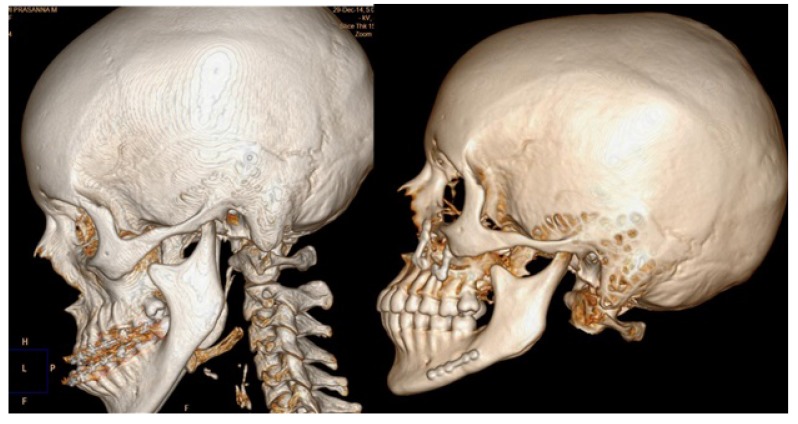
Pre and post-operative close up profile 3DCT picture showing bony window.

Three dimensional computed tomography (3DCT) scan data obtained from the sleep apnea study center as pre and post-operative data for analysis of all involved patients which have been taken one week prior to surgery and post-operatively after one year for sleep apnea assessment. Two angular variables and one linear variable were measured (Figs. **1** & **2**). 3D scans were performed with a Phillips Brilliance iCT 256 slice whole-body CT scanner by Gregard Phillips Amsterdam, Netherlands. Each patient required forty seconds of exposure for one scan. Voxel size was set at 0.45 mm for the sagittal, coronal and axial images and each scan contained 555 slices with bony and soft tissue reconstructive images. Each data set was imported directly into Dicom^®^ PMS D view (Figs. **1** & **2**). The scans were analyzed and the linear (in millimeters) and angular (in degrees) measurements were done *via* Dicom software. Frontal view and Profile view of subjects were measured in pre and post-operative 3DCT. Other relevant figures like pre and post-operative patients photographs (Fig. **3**), lateral cephalograms (Fig. **4**) and three dimensional computed tomography bony window (Fig. **5**) are also illustrated.

### 2.3 Statistical Analysis

The observations were tabulated using Microsoft Excel (Table **1**). Descriptive statistics were used to interpret the data. All statistics were calculated using SPSS *ver*. 20.0 with mean, SD, and range for analysis.

## 3 RESULTS

Changes in the nasolabial angle after maxillary advancement: Two patients underwent maxillary advancement of 4 mm and 5mm in which the nasolabial angle was increased by 5.6° and 10.7°, respectively. So, the mean advancement in the maxilla was 4.5mm and the mean difference was 8.15°. Hence, 1mm forward movement of the maxilla is an increase in the nasolabial angle by 1.81° (Table **2**).

## 3.2 Changes in Nasolabial Angle After Maxillary Setback

A total of five patients have undergone maxillary setback of 2 mm to 3 mm in which the nasolabial angle has decreased by 4.1 to 11.5°, respectively. So, the mean setback in the maxilla was 2.6 mm and the mean difference was 7.12°. Hence, 1mm movement of maxilla setback is a decrease in the nasolabial angle by 2.73° (Table **2**).

## 3.3 Changes in Mentolabial Angle after Mandibular Advancement

Among six patients, three patients underwent mandibular advancement of 2 mm to 8 mm. In three patients, the mentolabial angle was decreased by 7.6° and in remaining three patients, mentolabial angle increased by 3.6° (Table **3**).

## 3.4 Changes in the Mentolabial Angle After Mandibular Setback

Four patients underwent mandibular setback of 2 mm to 9 mm in which mentolabial angle decreased by 3.27° with 1mm mandibular backward movement (Table **3**).

## 3.5 Changes in the Lip Width in Bi Jaw Surgeries

Four patients underwent maxillary setback and mandibular advancement in which the mean maxillary setback was 2.75 mm. and the mean mandibular advancement was 3.0 mm. The mean decrease in the lip width was 2.15 mm. Three patients were operated for single jaw surgery (mandible) and two patients underwent maxillary advancement and mandibular setback showed no significant relationship with change in lip width. One patient with the setback of both the jaws had a decrease in the lip width. There was a significant relationship between lip width change only in the maxillary setback and mandibular advancement surgery when operated together (Table **4**).

## 4 DISCUSSION

The change in soft tissue morphology after surgical therapy is dependent on several factors like wound closure, lip morphology and post-operative swelling [8]. Assessment of soft tissue changes after surgical procedure requires minimum 6 months [9] to maximum 12 months [10]. Due to swelling, tissue redistribution, and functional adaption, long-term follow up is needed. The morphology of lip is also one of the determining factors [11]. Thick lips absorb a huge amount of bony advancement without any change in the soft tissue measurements. Dead space under the lip may absorb the first position of bony advancement before the soft tissue is affected in severe maxillary retrognathia [11].

Nasolabial angle is used to know the protrusion and retrusion of the maxilla in conjunction with the upper lip. It also helps in diagnosing the nasal tip projection. Nasal tip projection varies according to race, ethnicity, age and gender. Our study showed decreased nasolabial angle by 4.1° to 11.5° in maxillary setback of 2 mm to 3 mm (Table **2**). similar to Rosen HM [11] where 12 patients after moving the maxilla anteriorly and superiorly with follow up of 9.8 months concluded that at least 12 months are required before all residual edema to dissipate and complete animation of upper lip to return. If maxilla is moved superiorly without anteroposterior movements, the upper lip comes forward and nasolabial angle becomes more acute and conversely, if the maxilla is moved downward, the lip moves posteriorly and nasolabial angle becomes more obtuse. The study showed 0.51:1 ratio change in nasolabial angle after maxillary advancement. The mean setback in the maxilla was 2.6mm and the mean difference was 7.12° in our study which showed a decrease in the nasolabial angle by 2.73° with 1mm maxillary setback which clearly indicates that the impaction of the maxilla is causing the decrease in nasolabial angle and making the nasolabial angle more acute post-operatively. In Patrick J Louis study [12], maxillary advancement with a Le Fort I osteotomy (8 ± 2.5 mm) with eight months of follow up showed a decrease in the nasolabial angle by 5° (-10° to +7°). But our study results are in contrast to this study; as we operated only 2 patients. These patients underwent maxillary advancement of 4 mm and 5 mm in which the nasolabial angle is increased by 5.6° and 10.7°, respectively. So, the mean advancement found in the maxilla was 4.5 mm and the mean difference in nasolabial angle was 8.15°. Our study showed that 1mm forward movement of the maxilla, there is an increase in the nasolabial angle by 1.81°. Our results are supported by a study of Takahiro Shoji [13] where they found an increase in the nasolabial angle and projection of the nasal tip after maxillary advancement. Our results were obtained after 12 months whereas Rosen and Patrick assessed the changes after 9.8 months and 8 months, respectively. So, our results are more settled as suggested by Rosen HM for a minimum of 12 months follow up for the changes.

Mentolabial angle is influenced by the position of lower lip, chin and inclination of mandibular incisor teeth. An acute mentolabial angle may be a reflection of the dentoalveolar protrusion or an over-grown chin and in contrast, the obtuse mentolabial angle is because of dentoalveolar reclination or an undergrown chin [14]. Young –Kyun Kim *et al.* evaluated perioral soft tissue changes in 15 patients after mandibular setback surgery and found significant lip changes after 6 months of follow up. Lower lip protrusion was seen about 1.67 mm, soft tissue point B around 1.28 mm and pogonion around 1.61 mm [14]. Our study showed 2 mm to 9 mm of mandibular setback whereas, 8.1° to 23.5° decrease in mentolabial angle. So, the mean setback in the mandible was 4.75 mm and the mean difference was 15.57°. The mandibular setback of 1 mm showed a decrease in the mentolabial angle by 3.27° which made the mentolabial angle acute (Table **3**).

The increase of nasolabial angle and decrease of mentolabial angle were in accordance with previous study results [15-17]. Our study showed an increase in mentolabial angle in 3 patients and decrease in 3 patients, which were in significant relationships with the movement of the hard issue but not significant in relation to the procedure performed. The mandibular movement in our study was 2 mm to 4 mm whereas a decrease in mentolabial angle was 14.2° to 24.6°. The mean advancement in the mandible was 2.6 mm and the mean difference of mentolabial angle was 19.8°. Hence, 1 mm forward movement of mandible showed 7.6° decrease in mentolabial angle. The patients who underwent mandibular advancement of 4 mm to 8 mm showed an increase of mentolabial angle by 10.1° to 19.3° with 5.6 mm mean advancement and 20.2° mean difference in mentolabial angle. Hence, 1 mm forward movement of mandible showed an increase in the mentolabial angle by 3.6°.

Lip width is equal to the interpupillary distance in a normal individual. Yu Jin Jung [6] evaluated the hard and soft tissue changes in 17 subjects; where more soft tissue changes are related to horizontal and anteroposterior aspects than in the vertical one using 3DCT. The changes in the lip width were also not significant with the various procedures of orthognathic surgery. But in our study four patients underwent maxillary setback (mean of 2.75 mm) and mandibular advancement (mean of 3 mm) which showed mean decrease in the lip width by 2.15 mm and single jaw surgery with mandible showed no significant correlation in the changes with lip width. (Table **4**).

Evaluation of three-dimensional images, reproducibility, accuracy and availability of computed tomography proved to be the most reliable tool [18]. Researchers also proved that the soft tissue starts adapting from third month [18] and it takes more than a year to give desired results post-operatively [19], So we followed our patients after 12 months. Combinations of soft tissue remodeling, tissue relocation, hard tissue relapse, weight loss, and weight gain are important parameters to be considered for evaluation of postoperative changes in facial soft tissues [20]. In our study, we have not correlated that facts but taken the weight and built into consideration.

The activity of muscles in motion and sometimes at rest is a prime factor which should be considered for the success of treatment. [**Video - 1**] The patient at rest shows no incisal show but normal speech will show uneven lip movements and incisal exposure and even gummy smile. If the lip framework is not assessed properly then the patient and surgeon will not be satisfied even with orthognathic surgery. In such patients dynamic, the facial expression should be assessed properly for successful treatment. As an adjunct to the surgical procedure neurotoxins like Botox can be injected into a predetermined area to prevent gummy smile and satisfaction of the patient. Such procedures are minimally invasive, effective and innovative which can be used as an adjunct for true vertical maxillary excess with a hypermobile lip. In our few cases Botox was useful to reduce incisal show even after maxillary superior impaction. These views have been supported by few of the published literature [21-23].

Three-dimensional computed tomography is an effective tool for investigating the 3D changes in hard and soft tissues simultaneously in terms of direction and amount of movement information that 2D radiographs and three-dimensional surface scanning systems cannot provide [20]. The reproducibility of landmarks is better with no superimposition of structures. Image quality is in high resolution. If a better algorithm to combine the 3D laser or optical surface scanning and computed tomography without distortion error is developed, it would be a great advancement for the clinical research and the analysis can be performed with a larger sample size. The dynamism of perioral soft tissues irrespective of hard tissue positioning is the prime consideration for successful outcome [21-23].

Our study sample size was limited to 10 patients in a single center. The study was not confined to a single procedure, even it didn’t consider morphological factors and ageing changes. Even though our study consisted ten patients in a single center, we did 12 months follow up as recommended by published literature which is not available till date. Our study stands alone in this aspect. The study was not confined to the single procedure and it did not consider morphological factors and ageing changes. Our study suggests multicenter randomized control trials with long-term follow up to incorporate age changes.

## CONCLUSION

The amount of maxillary and mandibular advancement and setback plays an important role in the post-surgical increase and decrease in nasolabial angle, mentolabial angle, respectively. 3DCT scan and dynamic videography or clinical assessment are of paramount importance to assess the changes in perioral soft tissues where the published literature is scarce. The study also showed that conventional thought in nasolabial angle changes after surgery is changing because of underlying factors, hypermobility of the lip should be considered for prediction. Adjunctive therapy like Botox can be used to camouflage the hypermobility of lip for the success of treatment.

## Figures and Tables

**Table 1:** Cases records including procedure, movement and changes in perioral soft tissue changes Amo- anterior maxillary osteotomy, Sao - Su apical osteotomy, Bsso - Bilateral sagittal split osteotomy, Genio. - Genioplasty, Adv - Advancement, Sback - Setback, NLA- Nasolabial angle, MLA- Mentolabial angle, LW- Lip width, mm. - millimeter.

S.No.	Le fort		Amo		Bsso		Sao	Genio	Pre op NLA (°)	Post op NLA (°)	Pre op MLA (°)	Post op MLA (°)	Pre op LW (mm.)	Post op LW (mm.)
	Adv	Set back	Set back	Adv	Set back	Adv	Set back	Adv						
1	-	2mm	-	2mm	-	-	-	2mm	119.5°	109.6°	131.5°	106.9°	43.0	40.8
2	4mm	-	-	-	4mm	-	-	-	108.3°	113.9°	155.2°	139.9°	45.0	44.6
3	-	-	-	5mm	-	-	-	-	122.2°	123.1°	113.3°	132.6°	45.2	44.6
4	-	3mm	-	-	-	2mm	-	-	101.0°	96.7°	133.3°	119.1°	50.4	48.2
5	-	3mm	-	-	-	2mm	-	-	103.5°	99.4°	152.0°	131.8°	44.3	41.9
6	5mm	-	-	-	6mm	-	3mm	-	112.3°	123.0°	138.9°	130.8°	36.9	39.9
7	-	-	-	-	4mm	-	-	-	108.1°	108.6°	156.6°	133.1°	42.3	46.4
8	-	-	2mm	-	-	-	2mm	-	106.5°	100.7°	128.9°	113.5°	46.5	44.7
9	-	-	-	8mm	-	-	-	-	117.9°	118.9°	100.1°	131.3°	46.0	49.3
10	-	3mm	-	4mm	-	-	-	-	129.3°	117.8°	106.9°	117.0°	42.9	47.5

**Table 2:** Pre and post-operative differences in nasolabial angle after orthognathic procedures.

Procedure	Movement	Pre op	Post op	Difference
Max adv	4mm	108.3	113.9	Inc by 5.6
Max adv	5mm	112.3	123.0	Inc by 10.7
Max sback	2mm	119.5	109.6	Dec by 9.9
Max sback	3mm	101.0	96.7	Dec by 4.3
Max sback	3mm	103.5	99.4	Dec by> 4.1
Max sback	2mm	106.5	100.7	Dec by 5.8
Max sback	3mm	129.3	117.8	Dec by 11.5

Max – Maxillary, Adv – Advancement, Sback – Setback, Inc – Increase, Dec – Decrease, mm – millimeter, op- operative.

**Table 3:** Pre and post-operative differences in mentolabial angle after orthognathic procedures.

Procedure	Movement	Pre op	Post op	Difference
Mand adv	4mm	131.5	106.9	Dec by 24.6
Mand adv	2mm	133.3	119.1	Dec by 14.2
Mand adv	2mm	152.0	131.8	Dec by 20.7
Mand adv	5mm	113.3	132.6	Inc by 19.3
Mand adv	8mm	100.1	131.3	Inc by 31.2
Mand adv	4mm	106.9	117.0	Inc by 10.1
Mand sback	4mm	155.2	139.9	Dec by 15.3
Mand sback	9mm	138.9	130.8	Dec by 8.1
Mand sback	4mm	156.6	133.1	Dec by 23.5
Mand sback	2mm	128.9	113.5	Dec by 15.4

Mand – Mandibular, Adv – Advancement, Sback – Setback, Inc – Increase, Dec – Decrease, mm – millimeter, op- operative.

**Table 4:** Changes in lip width after orthognathic surgery.

Pt.no.	Maxilla	Mandible	Pre op	Post op	Difference
1	S 2mm	A 4 mm	43.0	40.8	Dec by 2.2
2	A 4 mm	S 4mm	45.0	44.6	Dec by 0.6
3	-	A 5 mm	45.2	44.6	Dec by 0.6
4	S 3mm	A 2 mm	50.4	48.2	Dec by 2.2
5	S 3mm	A 2 mm	44.3	41.9	Dec by 2.4
6	A 5 mm	S 9mm	36.9	39.9	Inc by 3.0
7	-	S 4mm	42.3	46.4	Inc by 4.3
8	S 2mm	S 2mm	46.5	44.7	Dec by 1.8
9	-	A 8 mm	46.0	49.3	Inc by 3.3
10	S 3mm	A 4 mm	42.9	47.5	Inc by 4.6

A – Advancement, S – Setback, Inc – Increase, Dec – Decrease, mm – millimeter, op- operative.
